# Arbuscular Mycorrhizal Fungi Increase the Phenolic Compounds Concentration in the Bark of the Stem of *Libidibia Ferrea* in Field Conditions

**DOI:** 10.2174/1874285801711010283

**Published:** 2017-10-31

**Authors:** Emanuela Lima dos Santos, Francineyde Alves da Silva, Fábio Sérgio Barbosa da Silva

**Affiliations:** 1 Post-graduation in Cellular and Applied Molecular Biology, Institute of Biological Sciences, University of Pernambuco, 310, Arnóbio Marques Street, Santo Amaro – 50100130 - Recife, Brazil; 2 University of Pernambuco, campus Petrolina, Laboratory of Mycorrhizal Technology (LTM/UPE)- Petrolina Center, BR 203, Km 2, 56328-900-Petrolina, Brazil; 3 University of Pernambuco, campus Santo Amaro, Laboratory of Mycorrhizal Technology (LTM/UPE) – Recife Center, 310, Arnóbio Marques Street, Santo Amaro – 50100130 - Recife, Brazil

**Keywords:** Caatinga, AMF, Bioactive compounds, Glomeromycota, Secondary metabolism, Field conditions

## Abstract

**Background:**

*Libidibia ferrea* is a species particular to the caatinga presenting medicinal properties for containing bioactive compounds. The use of Arbuscular Mycorrhizal Fungi (AMF) can increase the production of biomolecules in the legume leaves; however, no light has been shed on the role of symbiosis in maximizing metabolites production in the bark of *L. ferrea* stem.

**Objective:**

The aim was to select AMF that are efficient at increasing the production of phenolic compounds with medicinal properties in the bark of the *L. ferrea* stem.

**Methods:**

The experiment was designed in randomized blocks with four inoculation treatments (plants pre-inoculated with *Claroideoglomus etunicatum*, with *Gigaspora albida*, with *Acaulospora longula*, and non-inoculated plants – control) with six repetitions. Thirteen months after the transplanting, the plants were pruned and the bark of the stem was collected; subsequently, this plant material was dried in a chamber. After the drying process, fractions of the bark of the stem were macerated in methanol. The extracts were further used for analyses of the biomolecules.

**Results:**

The flavonoids concentration had an increase of, respectively, 236% and 186% in relation to the control for the treatments with *A. longula* and *C. etunicatum*; plants inoculated with *A. longula* had an increase of 47% in total tannins concentration compared with the non-inoculated control – a benefit that the proanthocyanidins did not present.

**Conclusion:**

Applying inoculation with *A. longula* may be an alternative to increase the production of biomolecules of the secondary metabolism in the bark of the *L. ferrea* stem in field conditions.

## 1 INTRODUCTION

*Libidibia ferrea* is a medicinal tree legume particular to the caatinga, popularly known as *pau-ferro* or *jucá* [1]. The population uses it in the treatment of diabetes, anemia, respiratory and gastrointestinal diseases [2] because of its therapeutic properties such as anti-inflammatory [3], antimicrobial [4], among others.

The medicinal action of *L. ferrea* is associated with the presence of secondary compounds in the phytomass found in different parts of the plant, such as flavonoids, phenols, tannins, and others [5]. Because of its therapeutic potential, *L. ferrea* is included in the Relação Nacional de Plantas Medicinais de Interesse ao Sistema Único de Saúde (Renisus - Brazil) [6].

The optimization in the production of these biomolecules may be achieved through the inoculation of Arbuscular Mycorrhizal Fungi (AMF) [5, 7, 8]. AMF are microorganisms belonging to phylum Glomeromycota [9] that form mutualistic symbiosis with the plants and colonize most of the studied angiosperms and gymnosperms, in addition to some pteridophytes and bryophytes [10].

When in symbiosis, the AMF improve the nutritional condition of plants [11, 12], promote their development [13], and increase the production of secondary compounds adding value to the plant phytomass, which may improve its therapeutic potential [14-17].

Some mechanisms are suggested to explain the accumulation of compounds in the secondary plant metabolism in response to mycorrhizal symbiosis as an improvement in the nutritional condition of the host [18, 19] as well as alterations in the activity of key-enzymes [20], activation of metabolic routes [21], increase in gene expression [21, 22], among others.

Few are the studies associating the production of secondary metabolites with inoculated plants established in the field [23-26]; among the existing studies, only one was conducted in Brazil, with Silva *et al*. [27] recording an increase in the foliar concentration of Gallic acid in mycorrhizal *L. ferrea*.

There are no records on the production of bioactive compounds in the bark of the *L. ferrea* stem in response to mycorrhizal inoculation. Considering that such part of the plant is highly used as therapeutic alternative, the aim of this study was to select AMF that are efficient at increasing the production of phenolic compounds with medicinal properties in the bark of the *L. ferrea* stem; in this context, we tested the hypothesis that inoculation with AMF increases the production of phenolic compounds in the bark of the *L. ferrea* stem; however, the benefits vary according to the inoculated AMF.

## 2 MATERIAL AND METHODS

We developed the experiment in February, 2013 at the Experimental Field of the Mycorrhizal Technology Laboratory (LTM/UPE), located at University of Pernambuco, *campus* Petrolina, BR 203, Km 2, Petrolina, PE- Brazil. The phytochemical and mycorrhizal assessments were carried out 13 months after the field transplanting.

### 2.1 Experimental Design

We conducted the experimental design in randomized blocks with four inoculation treatments (plants pre-inoculated with *C. etunicatum*, plants pre-inoculated with *G. albida*, plants pre-inoculated with *A. longula*, and non-inoculated plants – control) with six repetitions.

### 2.2 Arbuscular Mycorrhizal Fungi

We employed three AMF isolates: *Acaulospora longula* Spain & N.C. Schenck (UFPE 21), *Claroideoglomus etunicatum* (W. N. Becker & Gerdemann) C. Walker & A.Schussler) (UFPE 06), and *Gigaspora albida* N.C. Schenck & G.S. Sm. (UFPE 01) provided by the Mycorrhizae Laboratory of the Mycology Department of the Federal University of Pernambuco. The AMF were multiplied on soil with ten percent of vermicompost and *Panicum miliaceum* L. as the host; the inoculum was stored at 4 ºC until use.

### 2.3 Production of the *L. ferrea* Seedlings

We produced the *L. ferrea* seedlings using experimental screen, covering the period between July 2012 and February 2013. The seeds were germinated and transferred to pots with soil containing 1.2 Kg soil + five percent vermicompost when the plantlets presented two definitive leaves and subsequently inoculated or not with soil inoculum containing 200 spores, hyphae, and colonized roots from each AMF tested. After 225 days, the seedlings were transplanted into the field. At the transplanting, the seedlings presented in average 72 cm of height, 5.1 mm of stem diameter and 14 leaves as well as a colonization rate varying from 6.2% for the control plants to 53.45% for the treatments of inoculation.

### 2.4 Preparing the Experimental Area

Before the installation of the experiment, the area with 2,400 m^2^ was ploughed, gridded, and caved. Each cave (40 x 40 x 40 cm) was fertilized using five liters of vermicompost and 150 g of simple superphosphate; the irrigation was carried out using semi-automatic dripping (8.4 L H_2_O plant^-1^ h^-1^). The soil of the experimental field presented the following chemical characteristics at the depth of 0 – 20 cm: P 10.38 mg dm ^-3^, K 0.24 cmol_c_ dm^-3^, Ca 1.4 cmol_c_ dm^-3^, Mg 0.5 cmol_c_ dm^-3^, Na 0.03 cmol_c_ dm^-3^, Al 0.00 cmol_c_ dm^-3^, organic matter 0.41 g Kg^-1^, pH 6.2, electrical conductivity 0.21 mS cm^-1^.We transplanted 96 plants arranged in six blocks, consisted of four plants per treatment, considering two plants for analyses, resulting 16 plants per block. The plants were arranged in lines with a border around the experimental field with non-mycorrhizal *L. ferrea*.

### 2.5 Phytochemical Analyses

#### 2.5.1 Preparing the Plant Extract

Thirteen months after the beginning of the experiment, we pruned the plants and collected the bark of the stem; subsequently, we dried this plant material in a chamber (Biopar, Porto Alegre, RS, Brazil) (45 ºC) for three days. After the drying process, fractions of 500 mg of the bark of the stem were macerated in 20 mL methanol (70%, v/v) (F Maia, Cotia, Brazil) for ten days at 20 ºC [28]. The extracts were gauze-filtered, re-filtered in qualitative filter paper, and stored in amber flasks in a freezer.

#### 2.5.2 Flavonoids

For the quantification of flavonoids were transferred to a volumetric flask (25 mL): 1 mL methanolic extract, 0.6 mL glacial acetic acid (F Maia, Cotia, Brazil) and added 10 mL of a pyridine/methanol solution (2:8, v/v) (Vetec, Duque de Caxias, Brazil/ F Maia, Cotia, Brazil) and 2.5 mL aluminum chloride (5%, w/v) (Vetec, Duque de Caxias, Brazil) in methanol (F Maia, Cotia, Brazil). The volume was completed with distilled water and the solution was put to rest for 30 minutes; subsequently, we carried out a reading using a spectrophotometer (Biospectro, Curitiba, Brazil) (420 nm) with rutin (Sigma-Aldrich, São Paulo, Brazil) in the standard curve [29].

#### 2.5.3 Total Phenols

Total phenols were quantified according to the Folin-Ciocalteau method: 2 mL of the extract in volumetric flask (100 mL) added with five mL of the Folin-Ciocalteau reagent (10%, v/v) (Merck, Rio de Janeiro, Brazil) and 10 mL of sodium carbonate solution (7.5%, w/v) (F Maia, Cotia, Brazil). The volume was completed with distilled water and the solution remained at rest for 30 minutes. Subsequently, we conducted a spectrophotometric reading (760 nm) using tannic acid (Vetec, Duque de Caxias, Brazil) for the standard curve [30].

#### 2.5.4 Total Tannins

We carried out the quantification using modified casein precipitation method. We introduced three mL of the extract in an amber flask added with 0.5 g casein (Vetec, Duque de Caxias, Brazil); subsequently, the mixture was stirred for three hours (160 rpm) at 25 ºC. The samples passed through qualitative filter paper, the volume was inserted in a volumetric flask and completed until 25 mL with distilled water. We carried out the quantification of phenols for this solution using the Folin-Ciocalteau method [30]. The concentration of total tannins was obtained from the comparison of the difference of the values found in this analysis and the values found in the analysis of total phenols. We used tannic acid as standard curve (Vetec, Duque de Caxias, Brazil).

#### 2.5.5 Total Proanthocyanidins

Total proanthocyanidins were dosed through modified acid-vanillin method [31]. We introduced 10 µL of the extract in a test tube and 990 µL distilled water to dilute the extract as well as 2 mL vanillin solution (2%, w/v) (Vetec, Duque de Caxias, Brazil) in H_2_SO_4_ (70%, v/v) (F Maia, Cotia, Brazil). The solution remained at rest in the dark for 15 minutes; subsequently, we carried out a spectrophotometric reading (500 nm) using calibration curve prepared with catechin solution.

### 2.6 Mycorrhizal Analyses

#### 2.6.1 Mycorrhizal Colonization and Spores Density

For the mycorrhizal analyses, we collected soil at three equidistant points in the rhizosphere of the plants with depth between zero and 20 cm. For the mycorrhizal colonization, the roots were clarified with KOH (10%, w/v) and hydrogen peroxide (10%, v/v) (F Maia, Cotia, Brazil) as well as colored with Trypan blue (0.05 w/v, in lactoglycerol) (Vetec, Duque de Caxias, Brazil) according to method by Philips and Hayman [32]. We estimated the colonization percentage through the quarter intersect method [33]. The glomerospores were extracted from the soil using the wet sieving methodology, decantation [34], and centrifugation in water and sucrose (45%, w/v) proposed by Jenkins [35] and quantified in stereomicrocospe (40 x)

#### 2.6.2 Statistical Analysis

The data were subjected to ANOVA and the means compared using Tukey test (P < 0.05) with Assistat software (7.7).

## 3 RESULTS

We recorded an increase of 30% in the diameter of the root for the plants inoculated with *A. longula* and 35% for those associated with *G. albida* in relation to non-inoculated control (Table **1**).

The rate of mycorrhizal colonization did not differ among the treatments (Table **1**). A higher density of spores was recorded in the rhizosphere of inoculated plants with emphasis to the treatments with *A. longula* and *C. etunicatum,* presenting the highest densities (Table **1**).

Averages followed by the same letter do not differ from the Tukey test (P < 0.05).

The production of some compounds of the secondary metabolism in the bark of the *L. ferrea* stem was favored by mycorrhization with an increase in the concentration of flavonoids from 236% and 186%, respectively, in relation to the control, for the treatments with *A. longula* and *C. etunicatum* (Table **2**). In plants inoculated with *A. longula,* the concentration of total tannins was increased in 47% in relation to the control, a benefit that did not occur in the treatments with *G. albida* and *C. etunicatum* (Table **2**). The concentration of total phenols did not differ among the treatments; in contrast, the concentration of proanthocyanidins, in the plants associated with *A. longula* was lower than that recorded for the control (Table **2**).

## 4 DISCUSSION

Although the mycorrhizal colonization had indicated no differences between the treatments with inoculation and the control treatment (Table **1**), it is possible to assume that the inoculated AMF were more efficient than the native AMF, considering that they favored the increase in the root diameter of mycorrhizal plants (Table **1**). Similarly, a field study by Singh *et al.* [36] in semi-arid conditions recorded that the inoculation with *Rhizophagus fasciculatus* favored the development of *Coleus forskohlii* Briq. Other studies with legumes had also reported such benefit with the inoculated AMF favoring the increase of species and, in contrast with our study, producing more mycorrhizal structures than the control treatment [37]. However, in seedlings of *Inga vera*, legume occurring in the caatinga, mycorrhized with *A. longula*, *G. albida*, and *C. etunicatum* [38] as well as in inoculated *L. ferrea* established in the field [27], the inoculation with AMF did not result in difference in the root diameter in relation to the non-inoculated control. This benefit may be associated with the presence of arbuscules in the inoculated roots since such structure favors the exchange of nutrients in the roots [39], resulting in greater development.

In this study, the density of glomerospores in the plants mycorrhized with *A. longula* and *C. etunicatum* was higher than in the non-inoculated plants (Table **1**). Similar results were recorded in inoculated *C. forskohlii* in field conditions with the density of the spores in the mycorrhized plants presenting differences in relation to the plants in the control treatment without inoculation and containing vermicompost [25]. Silva *et al.* [27] recorded results that differ from our study considering that the density of the spores in the rhizosphere of *L. ferrea,* in field conditions, did not differ among the inoculation treatments. The higher production of these propagules in the soil guarantees new colonization sites in the *L. ferrea* since favorable conditions may cause the spores to germinate and colonize the host [40].

The study was pioneer at verifying an increase in the concentration of flavonoids and tannins in the bark of the stem due to mycorrhization, with emphasis to fungi *A. longula* (Table **2**). Similar results had been reported regarding leaves of *Pogostemon patchouli* Pellet, inoculated with native fungi presenting increase in the concentration of flavonoids and tannins [11]; in roots of *Glycyrrhiza glabra* L. a legume species, mycorrhization also increased the concentration of these compounds [37]; the shoots of mycorrhized *Viola tricolor* L. presented an increase in the concentration of flavonoid and rutin in relation to the non-inoculated control [41]. Seedlings of native legume species of caatinga biome also presented an increase in the concentration and foliar content of flavonoids and tannins [38, 42].

In contrast with the records in this study (Table **2**), Silva *et al.* [5] verified in greenhouse that the foliar concentration of tannins indicated no differences among the treatments. In *L. ferrea* established in the field, the foliar concentration of tannins presented no difference caused by mycorrhization [27], such records are different from ours considering that the inoculation provided increase in the concentration of tannins in the bark of the stem of *L*. *ferrea.*

These results suggest that the production of compounds of the secondary metabolism in response to the inoculation with AMF may vary in a single species according to the age of the plants, the part studied, and the type of experimental condition. Furthermore, the increase in the concentration of these biomolecules can be caused by the increase in the concentration of precursors of these compounds, such as the gallic acid, which can be optimized through mycorrhizal inoculation in *L. ferrea* [27]; in addition, the activity of key-enzymes such as chalcone synthase, which benefits the formation of flavonoids, can be increased with a few inoculated treatments [20].

In accordance with the results by Silva *et al.* [27], who studied the concentration of phenols in leaves of *L. ferrea*, seven months after the transplanting, the concentration of phenols in the bark of the stem did not differ among the treatments (Table **2**). In contrast, Riter Netto *et al.* [19] developed a study using screens and reported an increase in the foliar content of total phenols in *Passiflora alata* Curtis inoculated with *C. etunicatum*, *Rhizophagus intraradices*, and mixed inoculum (*Rhizophagus clarus* and *Gigaspora margarita*) in relation to the non-inoculated control. Other field studies that differ from ours indicate that inoculation with AMF provided an increase in these compounds, as recorded in flowers of *Cynara cardunculus* L. var. *scolymus* F. mycorrhized with *Funneliformis. mosseae* and *R*. *intraradices* [24] and plants of *Olea europaea* L. inoculated with *R. intraradices*, with an increase in the concentration of total phenols in their fruits and alterations in the phenolic composition of the oil extracted [26]. It suggest that the benefit of mycorrhization in the production of metabolites may vary according to the plant species used, the part of the plant studied, and the AMF.

Other studies on legumes from the caatinga inoculated with AMF reported an increase in the foliar concentration of phenolic compounds [38, 42]. Seedlings of cebil (*Anadenanthera colubrina* (Vell.) Brenan) had an increase in the concentration of phenols, flavonoids, and tannins due to the inoculation of mix (*G. albida* and *A. longula*) in relation to the non-inoculated control [42]. Oliveira *et al.* [8] also reported that seedlings of inoculated *Amburana cearensis* (Allemao) A. C. Smith presented increase in the foliar concentration of tannins, flavonoids and phenols, with emphasis to fungi *C. etunicatum*. It is possible to infer that the application AMF results in different benefits to the production of groups of phenolic compounds, which depends on the fungi species employed, corroborating the initial hypothesis of our study.

This study was pioneer at recording the production of proanthocyanidins in plants inoculated with AMF. The lower concentration of these biomolecules in plants mycorrhized with *A. longula* (Table **2**) is probably related to the accumulation of flavonoids in the plants of this treatment considering that some precursor molecules of the proanthocyanidins, with emphasis to the units flavan-3-ol, are also molecules involved in the biosynthesis of flavonoids [43, 44]. Therefore, we may infer that these precursors can have been used by the plant metabolism in the formation of flavonoids resulting in the lower concentration of proanthocyanidins verified.

The accumulation of biomolecules of the secondary plant metabolism in response to symbiosis may be attributed to mechanisms as the improvement of the nutritional condition of the host [18, 19], activation of metabolic routes [21], production of signaling molecules [20], alterations in the activity of key-enzymes for the production of these compounds [20], hormonal alterations [45], increase in the expression of genes involved in the biosynthesis of these biomolecules [22, 45]. Such mechanisms have also been suggested in other studies [8, 38].

The results obtained from this study indicate that some of the above mentioned mechanisms have probably led to the increase in the production of flavonoids and tannins. The inoculation increase the concentration of precursors of these biomolecules, such as the Gallic acid, precursor of certain tannins [43], which in this plant species had an increase reported in the leaves [27] and the chalcones, precursors of flavonoids [43]. Furthermore, the symbiosis may have activated the expression of genes coding for enzymes related to the production of these compounds, such as the Phenylalanine-ammonia-lyase (PAL) and Chalcone synthase (Chs) associated with the production of flavonoids, which presented an increase in our study (Table **2**) with a consequent improvement of the key-enzymes activity. Therefore, it is important to develop studies to quantify precursor molecules using, for example, High-efficiency Pressure Liquid Chromatography (HPLC), in order to analyze gene expression employing techniques such as real-time PCR. The use of biotechnological protocol considering the application of AMF may be an alternative to increase the production of biomolecules of the secondary metabolism in the bark of the stem of *L. ferrea*, which may add value to the phytomass to be commercialized by the industry of herbal medicines. Further studies on different plant parts, such as flowers, fruits are required.

## CONCLUSION

Inoculation with *A. longula* may be an alternative to increase the production of biomolecules of the secondary metabolism in the bark of the *L. ferrea* stem in field conditions.

## Figures and Tables

**Table 1:** Stem diameter, colonization, spore density, in *Libidibia ferrea* plants, in the field, inoculated or non-inoculated with arbuscular mycorrhizal fungi (AMF), 13 months after transplanting, in Petrolina, Brazil.

**Variable**		**Inoculation Treatment**				
		Control		*A. longula*	*C. etunicatum*	*G. albida*
Stem diameter (mm)		24.6 b		32.0 a	29.7 ab	33.2 a
Colonization (%)		90.7 a		92.3 a	92.2 a	90.5 a
Spore density (50 g soil^-1^)		42.0 b		57.5 a	55.2 a	38.2 b

**Table 2:** Concentrations of total phenols, total flavonoids, total tannins and total proanthocyanidins in the stem bark of *Libidibia ferrea*, in the field, inoculated or non-inoculated with arbuscular mycorrhizal fungi (AMF), 13 months after transplanting, in Petrolina, Brazil.

**Variable**	**Inoculation Treatment**			
	Control	*A. longula*	*C. etunicatum*	*G. albida*
Total phenolics (mg g plant^-1^)	2.01 a	1.96 a	1.98 a	1.98 a
Total flavonoids (mg g plant^-1^)	156.1 c	524.2 a	445.7 b	177.7 c
Total tannins (mg g plant^-1^)	1.35 b	1.98 a	1.12 b	0.46 c
Total proanthocyanidins (mg g plant^-1^)	0.67 a	0.05 b	0.18 ab	0.26 ab

Averages followed by the same letter do not differ from the Tukey test (P < 0.05).
